# Supplementary material for: Genetic Association between Farrowing Rates and Swine Leukocyte Antigen Alleles or Haplotypes in Microminipigs
Source: Cells. 2022 Oct 5;11(19):3138. doi: 10.3390/cells11193138 (PMC9563624; doi:10.3390/cells11193138)
Supplement: Supplementary file 1 [file cells-11-03138-s001.zip › cells-1909957-supplementary.pdf]

cells MDPI

**Table S1.** Amino acid pairwise distances among nine *SLA-1* alleles.

| | SLA-1*04:01 | SLA-1*05:01 | SLA-1*08:04 | SLA-1*08:05 | SLA-1*10:02 | SLA-1*11:04 | SLA-1*12:01 | SLA-1*13:01 | SLA-1*15:02 |
|---|---|---|---|---|---|---|---|---|---|
| SLA-1*04:01 | | | | | | | | | |
| SLA-1*05:01 | 0.1606 | | | | | | | | |
| SLA-1*08:04 | 0.2455 | 0.2469 | | | | | | | |
| SLA-1*08:05 | 0.2205 | 0.2452 | 0.1203 | | | | | | |
| SLA-1*10:02 | 0.2183 | 0.2156 | 0.2382 | 0.2463 | | | | | |
| SLA-1*11:04 | 0.1796 | 0.1980 | 0.2722 | 0.2446 | 0.1620 | | | | |
| SLA-1*12:01 | 0.1458 | 0.1898 | 0.2438 | 0.2487 | 0.2027 | 0.1819 | | | |
| SLA-1*13:01 | 0.0480 | 0.1489 | 0.2932 | 0.2557 | 0.2367 | 0.1869 | 0.1440 | | |
| SLA-1*15:02 | 0.1022 | 0.1430 | 0.2599 | 0.2325 | 0.2278 | 0.1818 | 0.1307 | 0.0798 | |

Amino acid pairwise distances among nine *SLA-1* alleles were indicated as the number of amino acid substitutions per site from the SLA-1 amino acid sequences that were analyzed by MEGA X program using the JTT matrix-based model with a gamma distribution [23,24]. Over all mean distance was 0.1972. 181 amino acids from α1 to a part of α2 domains of *SLA-1* alleles were referred from those registered in the DDBJ/EMBL/GenBank databases (*SLA-1*04:01*; AF464002: *SLA-1*05:01*; AF464044: *SLA-1*08:04*; AK396218: *SLA-1*08:05*; AF484015: *SLA-1*10:02*; DQ303230: *SLA-1*11:04*; EU440338: *SLA-1*12:01*; EU440335: *SLA-1*13:01*; EU440336 and *SLA-1*15:02*; AK232719).

**Table S2.** Amino acid pairwise distances among eight *SLA-3* alleles.

| | SLA-3*01:01 | SLA-3*03:05 | SLA-3*04:01 | SLA-3*05:02 | SLA-3*06:01 | SLA-3*06:02 | SLA-3*07:01:02 | SLA-3*08:01 |
|---|---|---|---|---|---|---|---|---|
| SLA-3*01:01 | | | | | | | | |
| SLA-3*03:05 | 0.1603 | | | | | | | |
| SLA-3*04:01 | 0.0596 | 0.1569 | | | | | | |
| SLA-3*05:02 | 0.0925 | 0.0946 | 0.0988 | | | | | |
| SLA-3*06:01 | 0.0452 | 0.1435 | 0.0652 | 0.1014 | | | | |
| SLA-3*06:02 | 0.0518 | 0.1657 | 0.0794 | 0.1165 | 0.0384 | | | |
| SLA-3*07:01:02 | 0.0385 | 0.1428 | 0.0584 | 0.0872 | 0.0188 | 0.0318 | | |
| SLA-3*08:01 | 0.1966 | 0.2061 | 0.2148 | 0.2142 | 0.1975 | 0.2184 | 0.2038 | |

Amino acid pairwise distances among eight *SLA-3* alleles were indicated as the number of amino acid substitutions per site from the SLA-3 amino acid sequences that were analyzed by MEGA X program using the JTT matrix-based model with a gamma distribution [23,24]. Over all mean distance was 0.1178. 188 amino acids from α1 to a part of α2 domains of *SLA-3* alleles were referred from those registered in the DDBJ/EMBL/GenBank databases databases (*SLA-3*01:01*; DQ303224: *SLA-3*03:05*; AB828067: *SLA-3*04:01*; AF464011: *SLA-3*05:02*; AF464041: *SLA-3*06:01*; AF464010: *SLA-3*06:02*; DQ303227: *SLA-3*07:01:02*; AK232238 and *SLA-3*08:01*; AK234307).

**Table S3.** Amino acid pairwise distances among eight *SLA-2* alleles.

| | SLA-2*03:02 | SLA-2*04:02:02 | SLA-2*05:04 | SLA-2*06:03 | SLA-2*09:01 | SLA-2*10:01 | SLA-2*11:01:02 | SLA-2*16:01 |
|---|---|---|---|---|---|---|---|---|
| SLA-2*03:02 | | | | | | | | |
| SLA-2*04:02:02 | 0.2413 | | | | | | | |
| SLA-2*05:04 | 0.3206 | 0.2170 | | | | | | |
| SLA-2*06:03 | 0.2412 | 0.2282 | 0.3217 | | | | | |
| SLA-2*09:01 | 0.2529 | 0.2665 | 0.2680 | 0.2351 | | | | |
| SLA-2*10:01 | 0.1932 | 0.2614 | 0.3158 | 0.1570 | 0.2429 | | | |
| SLA-2*11:01:02 | 0.2557 | 0.1818 | 0.2440 | 0.2157 | 0.2517 | 0.2571 | | |
| SLA-2*16:01 | 0.2087 | 0.2583 | 0.3304 | 0.1207 | 0.1340 | 0.1363 | 0.1726 | |

Amino acid pairwise distances among eight *SLA-2* alleles were indicated as the number of amino acid substitutions per site from the SLA-2 amino acid sequences that were analyzed by MEGA X program using the JTT matrix-based model with a gamma distribution [23,24]. Over all mean distance was 0.2330. 188 amino acids from α1 to a part of α2 domains of *SLA-2* alleles were referred from those registered in the DDBJ/EMBL/GenBank databases (*SLA-2*03:02*; DQ104338: *SLA-2*04:02:02*; EU432087: *SLA-2*05:04*; AF464005: *SLA-2*06:03*; AB828064: *SLA-2*09:01*; AB828065: *SLA-2*10:01*; EU432084: *SLA-2*11:01:02*; DQ303222 and *SLA-2*16:01*; AK233069).

**Table S4.** Amino acid pairwise distances among eight *DRB1* alleles.

| | DRB1*04:03 | DRB1*06:01 | DRB1*07:01 | DRB1*08:01 | DRB1*09:01 | DRB1*10:01 | DRB1*11:03 | DRB1*14:01 |
|---|---|---|---|---|---|---|---|---|
| DRB1*04:03 | | | | | | | | |
| DRB1*06:01 | 0.2215 | | | | | | | |
| DRB1*07:01 | 0.1921 | 0.2186 | | | | | | |
| DRB1*08:01 | 0.1894 | 0.1994 | 0.1047 | | | | | |
| DRB1*09:01 | 0.1966 | 0.1728 | 0.1225 | 0.0545 | | | | |
| DRB1*10:01 | 0.2218 | 0.1771 | 0.1505 | 0.1053 | 0.1196 | | | |
| DRB1*11:03 | 0.1480 | 0.1786 | 0.1853 | 0.2046 | 0.2001 | 0.2287 | | |
| DRB1*14:01 | 0.0705 | 0.2039 | 0.1623 | 0.1825 | 0.1850 | 0.2149 | 0.1344 | |

Amino acid pairwise distances among eight *DRB1* alleles were indicated as the number of amino acid substitutions per site from the DRB1 amino acid sequences that were analyzed by MEGA X program using the JTT matrix-based model with a gamma distribution [23,24]. Over all mean distance was 0.1695. 144 amino acids from β1 to a part of β2 domains of *DRB1* alleles were referred from data registered in the DDBJ/EMBL/GenBank databases (*DRB1*04:03*; AF464037: *DRB1*06:01*; AF464033: *DRB1*07:01*; AY102481: *DRB1*08:01*; AF464034: *DRB1*09:01*; AF464050: *DRB1*10:01*; CJ030292: *DRB1*11:03*; AB828137 and *DRB1*14:01*; DQ303220).

**Table S5.** Amino acid pairwise distances among six *DQB1* alleles.

| | DQB1*03:03 | DQB1*04:01:02 | DQB1*04:02 | DQB1*05:01 | DQB1*05:02 | DQB1*06:01 |
|---|---|---|---|---|---|---|
| DQB1*03:03 | | | | | | |
| DQB1*04:01:02 | 0.0794 | | | | | |
| DQB1*04:02 | 0.0867 | 0.0057 | | | | |
| DQB1*05:01 | 0.0673 | 0.0686 | 0.0759 | | | |
| DQB1*05:02 | 0.0791 | 0.0748 | 0.0678 | 0.0235 | | |
| DQB1*06:01 | 0.0667 | 0.0837 | 0.0826 | 0.0677 | 0.0469 | |

Amino acid pairwise distances among six *DQB1* alleles were indicated as the number of amino acid substitutions per site from the DQB1 amino acid sequences that were analyzed by MEGA X program using the JTT matrix-based model with a gamma distribution [23,24]. Over all mean distance was 0.0651. 144 amino acids from β1 to a part of β2 domains of *DQB1* alleles were referred from data registered in the DDBJ/EMBL/GenBank databases (*DQB1*03:03*; AF464047: *DQ1B*04:01:02*; DQ303218: *DQB1*04:02*; AK397263: *DQB1*05:01*; AY133574: *DQB1*05:02*; *AY102477* and *DQB1*06:01*; AF464029).
